# Evaluating the relationship between serum immunoglobulin G (IgG) and A (IgA) anti-CagA antibody and the *cagA* gene in patients with dyspepsia

**Published:** 2017-04

**Authors:** Hashem Fakhre-Yaseri, Ali Baradaran-Moghaddam, Mehdi Shekaraby, Hamid Reza Baradaran, Seyed Kamran Soltani-Arabshahi

**Affiliations:** 1 Gastroenterology, Research Center for Gastroenterology and Liver Diseases, Firoozgar Hospital, Iran University of Medical Sciences, Tehran, Iran; 2 Department of Internal Medicine, Firoozgar Hospital, Iran University of Medical Sciences, Tehran, Iran; 3 Department of Microbiology, School of Medicine, Iran University of Medical Sciences, Tehran, Iran; 4 Immunology Research Center, Iran University of Medical Sciences, Tehran, Iran; 5 Department of Immunology, School of Medicine, Iran University of Medical Sciences, Tehran, Iran; 6 Department of Epidemiology, Iran University of Medical Sciences, Tehran, Iran

**Keywords:** *cagA*, IgG anti-CagA, IgA anti-CagA, *Dyspepsia*

## Abstract

**Background and Objectives:**

The cytotoxin-associated gene (*cag*) pathogenicity island is reported to be a major virulence factor of *Helicobacter pylori* infection. It is previously reported that the *cagA*-positive strains are more virulent, so it can be postulated that the *cagA*-positive gastritis will be more severe and the serum immunoglobulin G (IgG) and A(IgA) anti-CagA antibody titer will be higher. The aim of this study was to compare the relationship between IgG and IgA anti-CagA antibody and the *cagA* gene expression in patients with dyspepsia. Serum samples obtained from 130 dyspeptic patients with positive *H. pylori* in histological and Geimsa staining were tested for serum IgG and IgA anti-CagA antibody using the enzyme-linked immunosorbent Assay. The expression of the *cagA* gene was determined using PCR on the biopsy samples, taken via endoscopy.

**Results:**

In our material, the sensitivity of IgG anti-CagA antibody in identifying patients with a proven infection with the *cagA*-positive strains was 97.67%, and the negative likelihood ratios was 0.06. There was not significant correlation between serum IgA anti-CagA and the expression of the *cagA* gene among the dyspeptic patients.

**Conclusion:**

The IgG antibody titer was significantly higher in our patients with the *cagA*-positive *H. pylori* strain. However, in daily practice, the level of the IgG antibody titer cannot predict whether or not an individual carries a *cagA*-positive *H. pylori* strain, because there is a major overlap in the IgG antibody titer between the *cagA*-positive and *cagA*-negative patients.

## INTRODUCTION

*Helicobacter pylori* is a Gram-negative, microaerophilic bacterium that colonizes more than half of the world’s human population (1). Most individuals harboring *H. pylori* remain asymptomatic, but the presence of this organism is a risk factor for the development of peptic ulceration, gastric mucosa-associated lymphoid tissue lymphoma, and gastric adenocarcinoma (2, 3). Several virulence factors of *H. pylori* have been frequently associated with the most serious clinical outcomes. These include the cytotoxin-associated gene (*cag*) pathogenicity island, which encodes a protein that is injected into the cytoplasm of the host cell and induces cellular morphologic alterations, proliferation, adhesion, and apoptosis (3, 4). The *cagA* gene is a marker for the presence of the *cag* pathogenicity island (5). The prevalence rates of the *cagA* gene in some developing and developed countries are reported to be as high as 60% and 90%, respectively (6). In addition, the prevalence rate of the *cagA* in Iranian patients with dyspepsia is reported to be 68.5% (4). Given that the *cagA*-positive strain is more virulent, it can be postulated that the degree of inflammation will be more severe leading to a higher immunoglobulin G (IgG) antibody titer in patients with the *cagA*-positive *H. pylori* colonization (7).

Several serological studies using the Enzyme-Linked Immunosorbent Assay (ELISA) or Western Immunoblotting (WB) have been performed to detect antibodies against the *cag* antigens. The ELISA is widely used for the detection of antibodies in epidemiological and post-treatment studies. This serological test enables the detection of antibodies against specific *H. pylori* antigens such as the *cagA* and vacuolating cytotoxin gene A (*vacA*) antigens. More recently, the recombinant *cagA* has been used as an antigen in the ELISA for the specific serological diagnosis of *H. pylori* infection in patients with peptic ulcer disease and non-ulcer dyspepsia (8, 9).

In the present study, we sought to detect specific IgG and IgA responses against the CagA of *H. pylori* in 130 patients with dyspepsia. Additionally, we tried to determine whether serum antibodies to CagA accurately reflect the characteristics of infecting *H. pylori* strains with *cagA* gene positive and to test the hypothesis that serum IgG and IgA have enough accuracy to select patients for endoscopy and gastric biopsy.

## METHODS

**Fig. 1.**
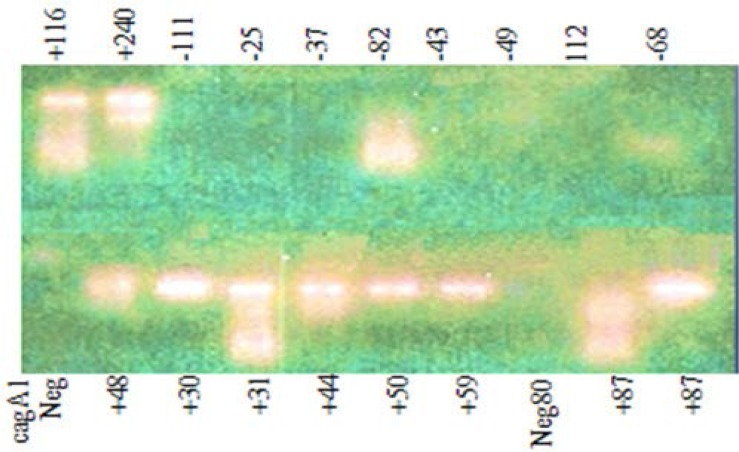
Cytotoxin-associated gene A-Positive and Negative Samples. Numbers indicate the code of each patient.

In the present study conducted between September 2011–2012, 130 dyspeptic patients with positive *H. pylori* in histology and the chief complaint of dyspepsia of over 2 months duration referring to the endoscopy ward of Firoozgar Hospital were selected. According to Leeds Medical School criteria (10), 802 patients, aged between 15 and 65 years, were selected. All the patients provided informed consent and accepted to complete a standard questionnaire. Esophagogastroduodenoscopy was done for all the patients in the same center by expert endoscopists. Among these subjects, 130 *H. pylori* infected patients with dyspepsia, with or without peptic ulcers, were selected and included in the study. The eligibility of the patients was based on the results of the questionnaire and esophagogastroduodenoscopy. Those patient who had no history of proved ulcer, previous *H. pylori* eradication, cigarette smoking, malignancy, and other underlying diseases in esophagogastroduodenoscopy, and nor did they use proton pump inhibitors or antibiotics (from at least 2 months before endoscopy) were excluded. Four biopsy specimens were taken from the antrum and gastric body for histological study and *H. pylori* detection. All the formalin embedded specimens were fixed and stained with Hematoxylin and Eosin Stain (H&E) or Geimsa. The specimens were evaluated by an experienced pathologist. A patient was considered to be positive for *H. pylori* when at least 5 bacilli in each microscopic field were found. Five ml of the blood sample was taken and sent to the immunology laboratory for anti-CagA antibody measurement. The genomic DNA was extracted from the biopsy samples using a DNA isolation kit for cells and tissues (Roche Applied Science Company) in according to the manufacturer’s instruction and stored at −20°C. Two sets of primers were designed complementary to the sequence located within the conserved region of the gene primers: CagA1 and CagA2. The primers sequences and size are depicted in Table 1 (11). The amplified products were detected after agarose gel electrophoresis, and, a 340bp (base pair) and indicated the presence of the *H. pylori* CagA in the specimen, as is illustrated in Fig. 1. The identification of gene product belong to *H. pylori* was confirmed via the PCR for the *glmM*, a conserved gene formerly known as urea C specific for this bacteria (11). Antibody measurement was performed using the commercial ELISA kit (Diagnostic Bioprobes, Milano-Italy). The anti-CagA antibody in the patients’ sera based on the synthetic CagA coated microplates. The complex was detected by 4RP conjugated antisera, and the color that developed was measured in 450-nm filters prefiltered at 620 nm. The optical density (OD) of each well was measured, and the antibody level was calculated using a standard calibration curve. The method is both sensitive and specific (98%), and its diagnostic sensitivity and specificity are more than 98%. A value ≥ 5 Arb/ml was considered positive for the anti-CagA antibody.

**Table 1.** Characteristics of the primers used for the detection of the *cagA* gene

Amplified Region	Primer Designation	Primer Sequence	Product Size (bp)	Ref.
*glmM*	GlmM-F	5′-AAGCTTTTAGGGGTGTTAGGGGTTT-3′	294	11
	GlmM-R	5′-AAGCTTACTTTCTAACACTAACGC-3′		
*cagA1*	CAG1-F	5′-GAT AAC AGG CAA GCT TTT GAG G-3′	349	11
	CAG1-R	5′-CTG CAA AAG ATT GTT TGG CAG A-3′		
*cagA2*	CAG2-F	5′-TTG ACC AAC AAC CAC AAA CCG AAG-3′	1385	11
	CAG2-R	5′-CTT CCC TTA ATT GCG AGA TTC C-3′		

*cag*, cytotoxin-associated gene; Bp, Base pair; *glmM*, urea C gene used for the detection of *H. pylori*; F, Forward; R, Reverse; CagA1 and A2, Two pairs of primers used for the detection of the Cag; ref, references

The data were analysed using SPSS package (version18) after encoding for each subject. Age is reported as age ± standard deviation (SD). The serum anti-CagA antibody, IgG and IgA, and *cagA* gene were compared, and the sensitivity, specificity, positive likelihood ratios [sensitivity /(1-specificity)], and negative likelihood ratios[(1-sensitivity)/specificity] of the serum anti-CagA antibodies were calculated for correlation. The results are presented with a confidence interval (CI) of 95%. Statistical significance was compared between the serum anti-CagA antibody, IgG and IgA, and *cagA* gene, detected by PCR (*cagA1*, *cagA2* positive or each one) using the Mantel-Haenszel chi-squared test with Yates correction or Fisher exact probability test. A P-value less than 0.05 was considered statistically significant

## RESULTS

According to the Leeds Medical School criteria, 802 patients had dyspepsia. The study population was comprised of 469 (58.5%) females and 333 (41.5%) males. The prevalence rate of *H. pylori* positive among these patients was 65.4%. Totally, 130 *H. pylori* infected patients, with ulcer and non-ulcer dyspepsia, were selected based on the results of a questionnaire and esophagogastroduodenoscopy. Of these patients, 43 (33.1%) and 87 (66.9%) had *cagA* gene positive and negative, respectively. The average age was 42.9 ± 12.1 (16 to 64) years in the *cagA* gene positive patients and 40.9±14.7 (16–64) years in patients with cagA gene negative. 21 (48.8%) and 45 (51.7%) of patients were female in *cagA* gene positive and negative patients, respectively (Table 2).

**Table 2.** Demographic information of 130 patients with dyspepsia based on positive and negative *cagA* gene

Findings	*cagA* genotypes		P-Value
	Positive (%)	Negative (%)	
Patients (n)	43 (33)	87 (67)	
Age (year)			
Mean age ± SD	42.9±12.1	40.9±14.7	0.12
Ranged	16–64	16–64	
Female (n)	21(48.8%)	45(51.7%)	0.77
Findings			
GU	11(25.6)	16(18.4)	-
DU	16(37.2)	22(25.3)	-
NUD	16(37.2)	49(56.3)	-

*cagA* ;Cytotoxin-associated protein A gene, n; number, SD; standard deviation, GU; gastric ulcer, DU; duodenal ulcer, NUD; nonulcer dyspepsia

The IgG anti-CagA antibody level ranged between 5 and 100Arb(Arbitrary)/ml. The mean±SD of the IgG anti-CagA antibody level in the patients with positive and negative *cagA* was 48.1±15.6 and 54.9 ±14.7, respectively. Also, the IgA anti-CagA antibody level ranged between 5 and 100Arb/ml. The mean± SD of the IgA anti-CagA antibody level in the patients with positive and negative *cagA* was 37 ± 9.4 and 51.5 ± 15.9, correspondingly (Table 3). The frequency rate of the positive serum IgG anti-CagA antibody was 96/130 (73.8%) in the entire study population and 42/43 (97.7%) in the patients with the *cagA*. Additionally, the frequency rate of the positive serum IgG anti-CagA antibody was 54/87 (62.1%) among the *cagA*-negative patients. According to these results, the sensitivity, specificity, positive likelihood ratios, and negative likelihood ratios of the positive serological results for the serum IgG anti-CagA antibody in the detection of the *cagA* gene were 97.96% (95% CI:86.20–99.87), 37.93% (95% CI:27.92–49.01), 1.57 (95% CI:1.32 1.86), and 0.06 (95% CI:0.00–0.44), respectively (Table 4). The frequency of the positive serum IgA anti-CagA antibody was 73/130 (56.1%) in the whole study population and 27/43 (62.8%) in the *cagA*-positive patients. Also, the frequency rate of positive serum IgA anti-CagA antibody was 46/87 (52.9%) in the *cagA*-negative patients. According to these results, the sensitivity, specificity, positive likelihood ratios, and negative likelihood ratios of the positive serological results for the serum IgA anti-CagA antibody in the detection of the *cagA* gene were 62.79% (95% CI:46.71–76.60), 47.12% (95% CI:36.44–58.07), 1.19 (95% CI:0.87–1.60), and 0.79 (95% CI:0.52–1.19), respectively (Table 4).

**Table 3.** Relationship between the Antibody Titer of IgG and IgA and the Presence of the CagA gene

Anti-CagA antibody		*cagA* genotypes			
Level (Arb/ml)		Positive (%)	Negative (%)
IgG	High (69–100)	15 (35.7)	22 (40.7)
	Medium(37–68)	10 (23.8)	17 (31.5)
	Low(5–36)	17 (40.5)	15 (27.8)
IgA	High (69–100)	6 (22.2)	20 (43.5)
	Medium(37–68)	2 (7.4)	5 (10.9)
	Low(5–36)	19 (70.4)	21 (45.6)

CagA- Cytotoxin-associated protein; *cagA*, Cytotoxin-associated protein A gene; IgG- Immunoglobulin G; IgA- Immunoglobulin A

**Table 4.** Accuracy of IgG and IgA Anti-CagA Antibody for diagnosis of *cagA* gene expression

**Anti-CagA** **Antibody**		***cagA* genotype**						
		**Positive (%)**	**Negative (%)**	**Sensitivity (%) (95% CI)**	**Specificity (%) (95% CI)**	**LR+ (95% CI)**	**LR-(95% CI)**	**P-Value**
**IgG**	Positive	42/43 (97.7)	54/87 (62)	97.67	37.93	1.57	0.06	0.009
	Negative	1/43 (2.3)	33/87 (38)	(86.20–99.87)	(27.92–49.01)	(1.32–1.86)	(0.00–0.44)	
**IgA**	Positive	27/43 (62.8)	46/87 (52.9)	62.79	47.12	1.19	0.79	0.39
	Negative	16/43 (37.2)	41/87 (47.1)	(46.71–76.60)	(36.44–58.07)	(0.87–1.60)	(0.52–1.19)	

CagA; Cytotoxin-associated protein A, IgG; Immunoglobulin G, IgA; Immunoglobulin A, CI; confidence interval, LR+; positive likelihood ratios, R; negative likelihood ratios.

## DISCUSSION

The determination of the IgG anti-CagA antibody may be a useful noninvasive method to identify infections caused by cytotoxic strains. In the present study, the sensitivity and the negative likelihood ratios of the serum IgG anti-CagA antibody in identifying patients with a proven infection with the *cagA*-positive strain were 97.67% and 0.06, respectively. In some of the cagA-negative patients, high levels of these antibodies were found, which is probably due to the immunological background of a previous *cagA*-positive *H. pylori* infection. In contrast to a previous study, our results demonstrated that the seropositive IgA anti-CagA antibody in the *cagA*-negative patients was higher than that in their *cagA*-positive peers (12). Although the IgA anti-CagA antibodies are more detected in in *cagA* gene negative patients (46 vs 41), in our study, but the difference was not significant, on the other hand the IgG level disappear for longer time after bacterial elimination, for such a reason 54 patients remain IgG positive when the *cagA* gene disappeared.

Serological experiments have shown that antibody titers with 120 to 128-kDa protein CagA correlate with the severity of the disease and that the CagA-producing strains are associated with dyspepsia (13). Chronic *H. pylori* infection elicits local and systemic immunological responses, leading to the production of IgA and IgG antibodies. Host B cells are activated by both Th1 and Th2 cells via specific cytokines and responses involving immunoglobulins. Perez-Perez et al. showed that nearly all of their subjects had IgG responses to the CagA, whereas fewer subjects had IgA responses to the whole cell antigen and, thus, concluded that IgG responses to *H. pylori* antigens were more universal than those of IgA responses (14). In general, the measurement of the serum IgG antibody level is preferred because the level of this antibody reflects the infection status more accurately. It is worth bearing in mind that IgG can be detected against cytotoxin-associated proteins. Cytotoxin-associated proteins are highly immunogenic and usually stimulate immune response and can be detected by serological methods such as the ELISA and WB. More recently, the recombinant *cagA* has been used as an antigen according to the ELISA for the specific serological diagnosis of *H. pylori* infection in patients with peptic ulcer and non-ulcer dyspepsia (9). The recombinant *cagA* is able to predict the expression of the CagA at protein level, obviating the need for sequencing. Azuma et al. reported that in their series of patients, some *H. pylori* strains possessed the CagA gene but were unable to produce the CagA protein and, thus, could be regarded as the *cagA*-negative strain (15).

The diagnosis of *H. pylori* infection usually involves esophagogastricdoudenoscopy (EGD) with biopsy since the only noninvasive method of comparable accuracy, the [13C] urea breath test, requires technical equipment that is not available in most care health centers. However, in recent years, serological methods for detection of *H. pylori* infection have reached sufficient accuracy to be used as screening tests before EGD. Most of the commercially ELISA system available for serological surveys are based on the detection of IgG antibodies against whole-cell preparations of *H. pylori.* Such preparations include antigens cross-reacting with other bacterial species, resulting in a specificity of these tests that rarely exceeds 90% (9). The test-and-treat strategy is a new option adopted in the recent decade by primary care physicians at primary care health centers, especially centers lacking endoscopic facilities. Talley and Vakil suggested that the test-and-treat strategy is preferable in populations with moderate to high prevalence of *H. pylori* infection (≥ 10%) (16). Accordingly, the IgG anti-CagA antibody may be used as a screening test instead of the conventional methods of measurement of anti-*H. pylori* IgG antibodies which raised against crude antigen suspension to select high-risk patients admitted for dyspepsia without alarming signs for upper gastrointestinal endoscopy.

Nevertheless, additional investigations with greater numbers of patients are required to fully elucidate whether there is an association between the serum IgG and IgA anti-CagA antibody and the *cagA*-positive strain. Moreover, some patients are infected with more than one type of bacteria possessing a variety of different proteins encoded by different genotypes of virulence genes.

In conclusion, our results suggest that a simple serological test such as ELISA could be helpful in identifying subjects infected with *H. pylori* strains producing *in vivo* major bacterial virulence factors possibly involved in patients with dyspepsia. The use of a specific anti-*H. pylori* antibody assay, especially for the detection of a specific serum IgG against the *cagA* antigen, may help to better select patients for endoscopy and gastric biopsy. The measurement of serum IgG anti-CagA antibody levels may be useful to select for upper endoscopy and to evaluate the eradication of the *cagA*-positive strain.
